# Analysis of *Escherichia coli* O157 strains in cattle and humans between Scotland and England & Wales: implications for human health

**DOI:** 10.1099/mgen.0.001090

**Published:** 2023-09-06

**Authors:** Margo Chase-Topping, Timothy J. Dallman, Lesley Allison, Nadejda Lupolova, Louise Matthews, Sonia Mitchell, Christopher J. Banks, Jamie Prentice, Helen Brown, Sue Tongue, Madeleine Henry, Judith Evans, George Gunn, Deborah Hoyle, Tom N. McNeilly, Stephen Fitzgerald, Alison Smith-Palmer, Sharif Shaaban, Anne Holmes, Mary Hanson, Mark Woolhouse, Xavier Didelot, Claire Jenkins, David L. Gally

**Affiliations:** ^1^​ The Roslin Institute and Royal (Dick) School of Veterinary Studies, University of Edinburgh, Edinburgh EH25 9RG, UK; ^2^​ Gastrointestinal Bacteria Reference Unit, Public Health England, London NW9 5HT, UK; ^3^​ Scottish *E. coli* O157/STEC Reference Laboratory, Royal Infirmary of Edinburgh, Edinburgh EH16 4SA, UK; ^4^​ Institute of Biodiversity, Animal Health & Comparative Medicine, University of Glasgow, Glasgow G12 8QQ, UK; ^5^​ Epidemiology Research Unit, Scotland’s Rural College, Inverness IV2 5NA, UK; ^6^​ Moredun Research Institute, Pentlands Science Park, Penicuik EH26 0PZ, UK; ^7^​ Public Health Scotland, Glasgow G2 6QE, UK; ^8^​ Usher Institute, University of Edinburgh, Edinburgh EH9 3DL, UK; ^9^​ School of Life Sciences and Department of Statistics, University of Warwick, Warwick CV4 7AL, UK

**Keywords:** *E. coli *O157, STEC, phage type, whole genome sequencing

## Abstract

For the last two decades, the human infection frequency of *Escherichia coli* O157 (O157) in Scotland has been 2.5-fold higher than in England and Wales. Results from national cattle surveys conducted in Scotland and England and Wales in 2014/2015 were combined with data on reported human clinical cases from the same time frame to determine if strain differences in national populations of O157 in cattle could be associated with higher human infection rates in Scotland. Shiga toxin subtype (Stx) and phage type (PT) were examined within and between host (cattle vs human) and nation (Scotland vs England and Wales). For a subset of the strains, whole genome sequencing (WGS) provided further insights into geographical and host association. All three major O157 lineages (I, II, I/II) and most sub-lineages (Ia, Ib, Ic, IIa, IIb, IIc) were represented in cattle and humans in both nations. While the relative contribution of different reservoir hosts to human infection is unknown, WGS analysis indicated that the majority of O157 diversity in human cases was captured by isolates from cattle. Despite comparable cattle O157 prevalence between nations, strain types were localized. PT21/28 (sub-lineage Ic, Stx2a+) was significantly more prevalent in Scottish cattle [odds ratio (OR) 8.7 (2.3–33.7; *P*<0.001] and humans [OR 2.2 (1.5–3.2); *P*<0.001]. In England and Wales, cattle had a significantly higher association with sub-lineage IIa strains [PT54, Stx2c; OR 5.6 (1.27–33.3); *P*=0.011] while humans were significantly more closely associated with sub-lineage IIb [PT8, Stx1 and Stx2c; OR 29 (4.9–1161); *P*<0.001]. Therefore, cattle farms in Scotland were more likely to harbour Stx2a+O157 strains compared to farms in E and W (*P*<0.001). There was evidence of limited cattle strain migration between nations and clinical isolates from one nation were more similar to cattle isolates from the same nation, with sub-lineage Ic (mainly PT21/28) exhibiting clear national association and evidence of local transmission in Scotland. While we propose the higher rate of O157 clinical cases in Scotland, compared to England and Wales, is a consequence of the nationally higher level of Stx2a+O157 strains in Scottish cattle, we discuss the multiple additional factors that may also contribute to the different infection rates between these nations.

## Data Summary

FASTQ files have been submitted to the National Center for Biotechnology Information (NCBI). All data can be found under Bioproject no. PRJNA315192. Individual accessions and isolate meta-data are provided in a separate file (Supplementary_material_accession_16012023.xls, available in the online version of this article).

**Impact Statement**

Based on yearly reports from public health authorities, it is evident that the rate of clinical *Escherichia coli* O157 infection is higher in Scotland than in England and Wales. *E. coli* O157 expressing Shiga toxin is a serious threat to human health with strains present in cattle as a major reservoir host. In Scotland we have been gathering data on cattle O157 since 1997. However, this is the first comprehensive snapshot of the strain composition in cattle and humans within Great Britain (England, Scotland and Wales) from the same time frame. The study has demonstrated the much greater diversity of *E. coli* O157 present in the surveyed cattle population in England and Wales compared with Scotland with the key finding that Stx2a-encoding PT21/28 strains, known to be associated with serious human disease, are more prevalent in Scottish cattle. We discuss whether this strain localization could be the main reason for the higher incidence of *E. coli* O157 infections in Scotland. This study has fostered collaboration among University and Public Health researchers working on a ‘One Health’ approach to food safety. The results of the survey represent a major advance in understanding how strain composition in the animal reservoir could impact the prevalence, incidence and burden of disease in humans.

## Introduction

Food-borne diseases represent a constant threat to public health and a significant impediment to socioeconomic development [1]. Worldwide, Shiga toxin-producing *Escherichia coli* (STEC) is an important cause of foodborne infections [1–3]. The incidence of STEC infections has been estimated to range from 0.6 to 136 cases per 100 000 patient years with one third caused by the serogroup *E. coli* O157 (O157) [2]. The total number of confirmed STEC infections was 3573 in 2009, increasing dramatically to 6 073 cases in 2017 [3]. Among the 31 countries in Europe, Germany and the UK had the highest human STEC infection rates [3]. Although STEC is a relatively rare human pathogen, presenting symptoms can include diarrhoea and bloody diarrhoea with haemorrhagic colitis, and haemolytic uraemic syndrome (HUS) as severe sequelae [4]. HUS develops in 10–15 % of cases, with the highest rates in people under 15 years or over 65 years of age [4].

There are three major lineages of *E. coli* O157 (O157) worldwide, I, I/II and II, which can be further divided into six sub-lineages (Ia, Ib, Ic, IIa, IIb, IIc) [5]. Further subtyping was dependent on the country and public health organization and included phage typing, pulsed-field gel electrophoresis and multiple locus variable-number tandem repeat analysis. More recently whole genome sequencing (WGS) has been adopted as the molecular typing method of choice for all STEC O157 isolates. The use of WGS-based typing analysis has been shown to improve trace back in the event of a food-borne outbreak, ensuring the rapid implementation of interventions to protect public health investigations [6]. Global adoption of WGS will facilitate worldwide comparison of strains. It is evident that specific subtypes of O157 are more likely to be associated with serious human disease and in part this is due to the different types of Shiga toxin (Stx) that can be produced by strains. There are two antigenically distinct types, Stx1 and Stx2, further subtyped into Stx1a,c,d and Stx2a-k [7, 8]. Clinical and public health risk assessment algorithms in many countries, including the UK, are based on using detection of Stx2 as a predictor of severe gastrointestinal disease [1, 5]. Strains of O157 with other *stx* subtypes may cause diarrhoea but their association with HUS is less certain and highly variable [5, 9]. In the UK, strains with Stx2a have been associated with more severe disease, especially HUS, than strains with Stx2c [5, 9–11]. The risk of severe illness, however, may also depend on virulence gene combinations and gene expression, dose ingested, and susceptibility of the human host.

*E. coli* O157 was first isolated in the UK in July 1983 [12] and remains the most commonly detected serogroup in clinical cases of disease [13, 14]. Over the last 25 years rates of clinical O157 cases have declined gradually, although the rate in Scotland remains consistently 2.5-fold higher than in England and Wales (Table S1; Fig. S1.1A). Healthy colonized cattle are considered the most important reservoir for O157 in humans in the UK although other ruminants especially sheep have been the identified source of human infections [15]. Zoonotic transmission occurs through consumption of contaminated food or water or from direct contact with infected animals or their environment. O157 can persist for long periods in the environment, including soil [16]. Large food-borne outbreaks caused by undercooked meat, dairy products, and raw vegetables and salads have been described [17]. A low infectious dose and propensity for person-to-person spread means transmission in households and closed settings such as schools can occur [18].

Previously, while there have been two national cattle STEC surveys conducted in Scotland [19], no comprehensive national cattle survey had ever been conducted in England and Wales. The Food Standards Agency and Food Standards Scotland funded a programme to examine the role of cattle as a source of human clinical O157 cases in Great Britain (GB) which included surveys of farms in Scotland and England and Wales with cattle destined for the food chain [20]. For the first time, the prevalence and strain characteristics of STEC O157 isolated from cattle and strains isolated from humans could be compared. We specifically aimed to address the question that if cattle are the main reservoir for reported human infections in GB, why do we observe such consistent differences in the rate of reported cases per 100 000 population between Scotland and England and Wales (Fig. S1.1A), given similar levels of O157 cattle prevalence between the two nations [21]. Our main hypothesis was that the nations may have strain types with different pathogenic potential for humans and such geographical variation combined with predominately local exposure may lead to the higher detected infection rate in Scotland.

## Methods

### Animal case data

Between September 2014 and November 2015, 110 farms in Scotland and 160 farms in England and Wales were selected for sampling as they were rearing beef cattle aged 1–2 years intended for the food chain. Some of the farms were mixed with both meat and dairy production. All samples were obtained from freshly voided faecal pats. The field sampling methodology has been published [20, 21] as well as analysis of associated metadata [22]. A farm was considered positive for O157 if at least one faecal pat was positive. From the 5676 samples collected, 521 O157 isolates from 60 positive farms were available for analysis in this study. Samples from England and Wales were combined for all analyses.

### Human clinical data

Faecal samples from patients presenting to healthcare settings with clinical symptoms suggestive of O157 infection, as well as asymptomatic contacts, were submitted to local diagnostic laboratories and cultured for the presence of non-sorbitol-fermenting (NSF) O157 (http://www.hpa-standardmethods.org.uk/). Local diagnostic laboratories sent presumptive isolates of STEC to the Gastrointestinal Bacterial Reference Unit (GBRU) at Public Health England (PHE) (England and Wales) and to the Scottish *E. coli* O157/STEC Reference Laboratory (SERL) (Scotland). All culture-positive human clinical cases for the same time frame as the cattle survey were identified by SERL and PHE. Cases considered to be overseas travel-related were excluded. For consistency with the animal data, human case data from England and Wales were combined prior to analysis.

### Typing data

#### Phage typing and stx subtyping

PCR (ISO/TS, 13136) was used to confirm the serogroup of the isolates as O157 and to test for the presence of Shiga toxin subtypes 1 (*stx_1_*) and 2a and 2c (*stx_2_*) and intimin (*eae*) using real-time PCR [19]. Phage typing was performed as previously described using a specific phage collection [18].

#### Whole genome sequencing

From human clinical cases, WGS was performed on 161 isolates from Scotland and 523 isolates from England and Wales. For Scotland this represented all presumed non-travel-related clinical samples, but for England and Wales this represented a random selection of non-travel-related, temporally dispersed isolates comprising approximately 60 % of all isolates received and typed as O157:H7 between September 2014 and November 2015. From cattle, WGS was carried out on 113 isolates from the 521 isolates collected across the 60 positive farms. The aim was to sequence at least one representative of each phage type/stx subtype isolated from each positive farm. So, for example, if there were eight PT21/28 Stx2a isolates, one PT21/28 Stx2a2c isolate and two PT32 stx2c isolates identified on one farm, then at least three isolates were sequenced. This was carried out to capture the diversity present on farms as defined by phage typing and stx subtyping. In addition all isolates were sequenced from six farms to look at their relatedness, with the assumption that strains of the same phage type (PT) on the same farm would be very closely related.

Genomic DNA from isolates of STEC was extracted on the QiaSymphony platform (Qiagen). The sequence library was prepared using the Nextera XT kit and sequenced on a HiSeq 2500 (Illumina) yielding paired-end reads of 100 bp in length. High-quality reads were mapped to the reference STEC O157 strain, Sakai (GenBank accession BA000007), using Burrows-Wheeler Aligner – Maximum Exact Matching (BWA MEM, v0.7.2) [23]. The sequence alignment map outputs from BWA were sorted and indexed to produce a binary alignment map (BAM) using Samtools (v1.1) [24]. Genome Analysis Toolkit (GATK v2.6.5) was then used to create a variant call format (VCF) file from each of the sorted BAMs, which were further parsed to extract only SNP positions of high quality [mapping quality (MQ) >30, depth (DP) >10, variant ratio >0.9]. Hierarchical single linkage clustering was performed on the pairwise SNP difference between all isolates at descending distance thresholds (Δ250, Δ100, Δ50, Δ25, Δ10, Δ5, Δ0) [6, 11]. SNP alignments were created tolerating positions where >80 % of isolates had a base call with regions of recombination masked using Gubbins v2.0.0 [25]. Maximum-likelihood phylogenies were computed using IQ-TREE v2.0.4 [26] with the best-fit model automatically selected and near-zero branches collapsed into polytomies. Shiga toxin subtyping was performed as previously described [27]. Lineage and sub-lineage assignments were performed based on discriminatory SNPs, extracted directly from SnapperDB v0.2.5, that define the population structure [28].

#### Clustering and analysis of whole genome sequence data

For each of the most frequently (>5 % prevalence) observed sub-lineages (Ic, IIa and IIc) present in both cattle and human clinical cases from England and Wales and Scotland the genetic distance was calculated between all pairs of isolates. The results were summarized using median distance and range. Probability density distributions of the genetic distances were calculated and differences in distributions assessed using Kolmogorov–Smirnov tests (SciPy v1.4.1). For both clinical isolates and animal isolates, genetically close matches (i.e. the closest 10 % of isolates within 50 SNPs) were extracted and the proportion of isolates from each location were compared to the overall proportions using a Fisher exact test (Python v3.7.6).

Ancestral state reconstruction was performed using BEAST v1.10.4 [29] using location of sampling as a discrete trait under a constant population size prior. The Markov chain Monte Carlo (MCMC) was run for 50 million iterations. Phylogenetic associations with location and host were investigated using TreeBreaker v1.1 [30].

#### Diversity profiles: phage type

The diversity of PTs for each location (Scotland vs England and Wales) and host (cattle vs human) combination was examined using Hill numbers [31], using a similar approach to that of Mather *et al*. [32]. This measure, Dq, can be used to calculate the effective number [33] of PTs, where q is a parameter that can be varied to control the extent to which rare PTs contribute towards calculated diversity. The following diversity measures were calculated for both surveys: species richness, D0; Shannon diversity, D1; Simpson diversity, D2; and Berger–Parker diversity, D∞ [32]. These analyses were conducted in R v3.0.2 (R Development Core Team, 2013), using rdiversity v2.0 [34].

#### Spatial distribution: phage type

Spatial differences in PT composition (cattle only) was examined by mapping distributions using QGIS (v3.4). In Scotland, past analysis has divided the country into six Animal Health Districts (AHDs) [19] including: Highland, Islands, North East, Central, South East, South West (see Fig. S1.2A). In England and Wales, spatial regions were defined using the Nomenclature of Units for Territorial Statistics (NUTS) including: North East, North West, Yorkshire, East Midlands, West Midlands, East of England, London, South East, South West, West. No farms were sampled in London, and therefore there were only results from nine regions for England and Wales (see Fig. S1.2B). Spatial regions were defined to ensure at least five farms in each region to preserve confidentiality.

#### Associations among typing methods

The distribution of PTs, stx subtype (Stx1a, Stx2a and Stx2c) and sub-lineage (Ia, Ib, Ic, IIa, IIb, IIc and I/II) were examined overall as well as across host (cattle/human) and location (Scotland/England and Wales) using either Chi-square or Fisher’s exact test as appropriate (StatXact v11). Fig. S2.1 illustrates how the typing methods compare using the WGS data generated in this study.

## Results

### Diversity of strain types in cattle

**Fig. 1.**
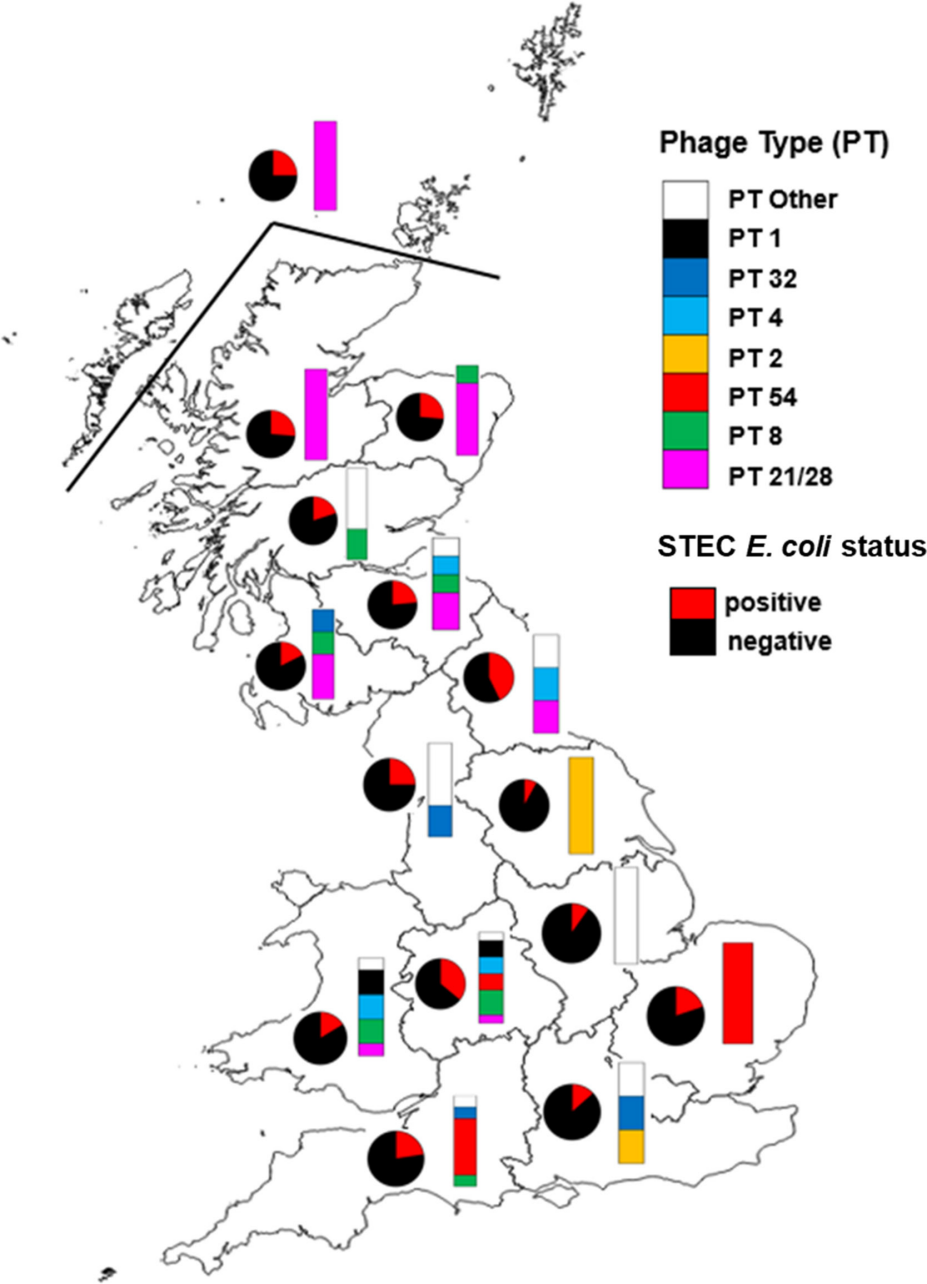
Cattle prevalence and spatial distribution of phage types (PTs). Map of the UK divided into regions. In Scotland, six Animal Health Districts (AHDs) include: Highland, Islands, North East, Central, South East and South West. In England and Wales, spatial regions were defined using the Nomenclature of Units for Territorial Statistics (NUTS). NUTS 1 regions for England and Wales include: North East, North West, Yorkshire, East Midlands, West Midlands, East of England, London (no data), South East, South West and West. Pie charts show the proportion of farms in the areas that were positive (red) and negative (black). Stacked bars show the proportion of all positive samples that were PT1, PT32, PT4, PT2, PT54, PT8, PT21/28 and PT Other (other includes the following PTs: 14, 31, 34, RDNC). Data associated with this figure are given in Table S5.1A&B. Spatial regions were defined to ensure at least five farms in each region to preserve confidentiality. European Parliament, Council of the European Union. Regulation (EC) No. 1059/2003 of the European Parliament and of the Council of 26 May 2003 on the establishment of a common classiﬁcation of territorial units for statistics (NUTS). *Ofﬁcial Journal of the European Union* L154 2003; 46 : 1.

The proportion of farms that were O157 positive was not significantly different when comparing Scotland with England and Wales. In Scotland the proportion of farms that were O157 positive ranged from 17.4 % (South West) to 29·4 % (South East) (*P*=0.9598) and within England and Wales from 8.3 % (Yorkshire) to 42.9 % (North East) (*P*=0.4135) (Fig. 1). The median number of isolates collected from each farm was 8.5 (range 1–44) and 3.0 (1–28) for Scotland and England and Wales respectively. On all farms approximately 90 % of the isolates were the same PT and stx subtype. For a few farms (*n*=6), WGS was performed on all sampled isolates including those with the same PT. Isolates with the same PT present on any of these specific farms showed negligible genetic distance (<5 SNPs) indicating they are very closely related.

Scottish cattle were significantly associated with the isolation of sub-lineage Ic strains [64.3 % vs 20 %, *P*<0·001, odds ratio (OR) 8.7 (2.38–33.7)] whereas cattle in England and Wales had a significantly higher isolation rate of sub-lineage IIa strains [17.1 % vs 10.7 %, *P*=0.011, OR 5.56 (1.27–33.3)] (Table 1).

**Table 1.** The proportion of isolates found in cattle and humans in Scotland and England and Wales from each sub-lineage for the same time frame as the cattle study

	Cattle				Human			
	Scotland		England and Wales		Scotland		England and Wales	
Sub-lineage	***n***	%	***n***	%	***n***	%	***n***	%
Ia	0	0	0	0	4	2.5	18	3.4
Ib	0	0	0	0	3	1.9	4	0.80
Ic	18	64.3	6	17.1	82	50.9	164	31.4
IIa	3	10.7	14	40.0	10	6.2	52	9.9
IIb	2	7.1	7	20.0	1	0.60	78	14.9
IIc	5	17.9	6	17.1	54	33.5	167	31.9
I/II	0	0	2	5.7	6	3.70	25	4.80
Other					1	0.60	15	2.90
Total	28		35		161		523	

**Fig. 2.**
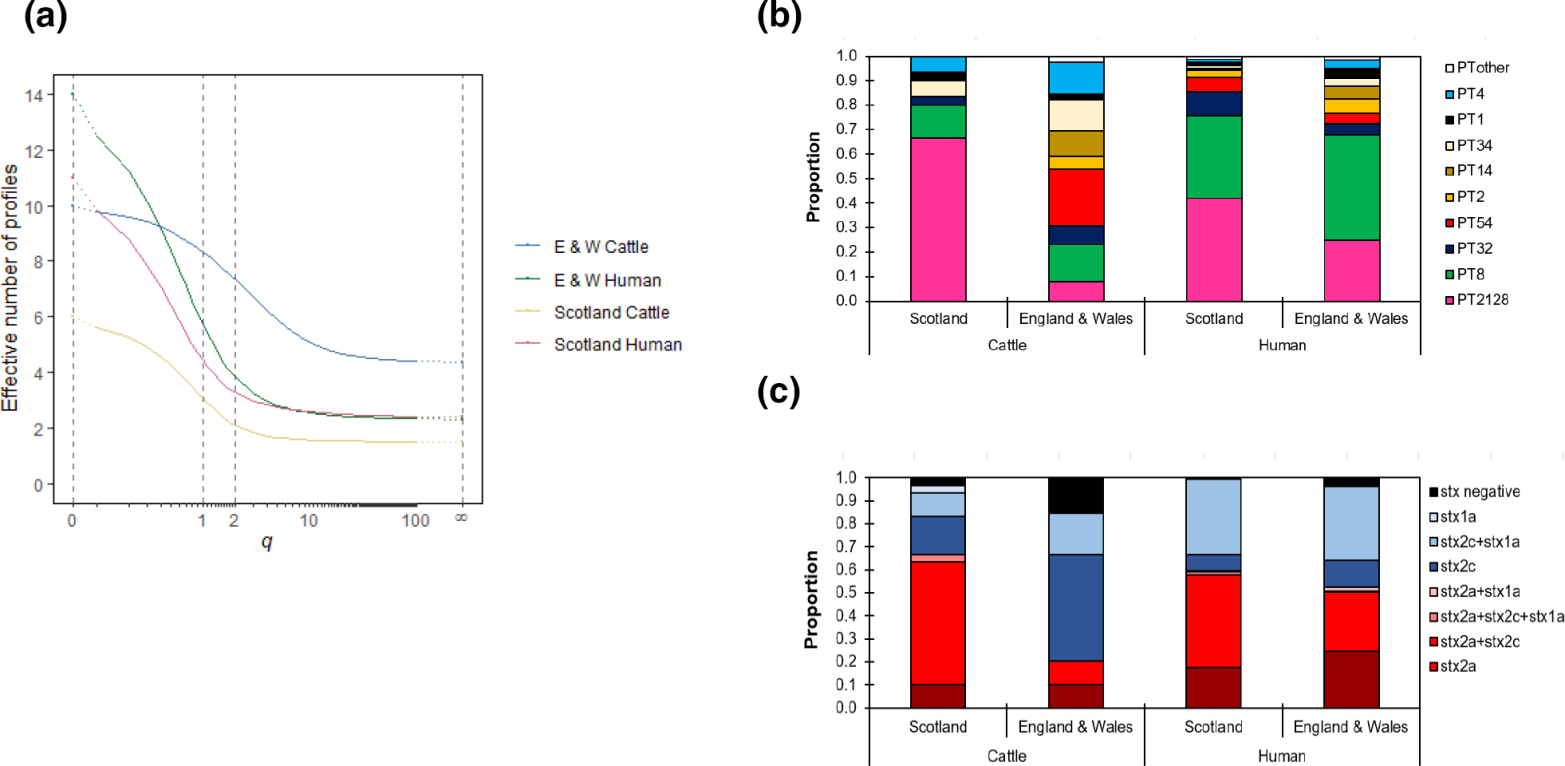
Diversity of cattle and clinical strains. Distribution of phage types (PTs) and stx subtypes for cattle and human clinical cases between September 2014 and November 2015. (**a)** Diversity profiles for cattle and human PTs from Scotland (cattle, yellow; human, red) and England and Wales (cattle, blue; human, green) illustrating the significantly lower diversity of PTs observed in Scottish cattle. Dq: the effective number of PTs; D0, Species richness; D1, Shannon diversity; D2, Simpson diversity; and D∞, Berger–Parker diversity. (**b)** Stacked bar chart showing the proportion of all isolates that were PT1, PT14, PT4, PT34, PT2, PT54, PT32, PT8, PT21/28 and PT Other in Scotland (cattle/human) and England and Wales (cattle/human). (**c)** Stacked bar chart showing the proportion of all isolates that were Stx2a, Stx2a+Stx2c, Stx2a+Stx2c+Stx1a, Stx2a+Stx1a, Stx2c, Stx2c+Stx1a, Stx1a or Stx negative in Scotland (cattle/human) and England and Wales (cattle/human).

The diversity of strains in Scottish cattle was significantly lower than that observed in cattle from England and Wales by every measure examined (Fig. 2a). Only six different designated PTs were recorded in Scottish cattle with PT21/28 comprising the majority of isolates. Scottish farms that were O157 positive were significantly more likely to have PT21/28 [*n*=20/26 (76.9%)) than farms in England and Wales [*n*=3/34 (8.8%)] [76.9 % vs 8.8 %; *P*<0.001; OR=34 (6.6–219)] (Fig. 2b). The most commonly isolated PT in English (only) cattle was PT54 [isolates: 79/234 (34 %) farms: 9/34 (26.5 %)]; this PT was not detected in Scottish cattle. Similarly, PT1 (England and Wales) and PT2 (England only) were not detected in cattle in Scotland.

Three main toxin *stx* subtypes were found in the sampled cattle: *stx1a, stx2a* and *stx2c* (Fig. 2c). Cattle farms in Scotland were more likely to harbour the *stx2a* variant compared to farms in England and Wales [66.7 % vs 20.5 %, *P*<0.001; OR 7.75 (2.33–26.6)].

There were spatial differences in cattle strain composition across Scotland and England and Wales, as highlighted in Fig. 1. In Scotland, northern farms were predominantly (80–100 %) PT21/28, whereas in England and Wales PT21/28 was only found in the North East, Wales and West Midlands (Fig. 1). The diversity of PTs varied across these parts of England and Wales, ranging from more than five PTs (e.g. West Midlands) to only one (e.g. Yorkshire).

### Analysis of cattle lineages

**Fig. 3.**
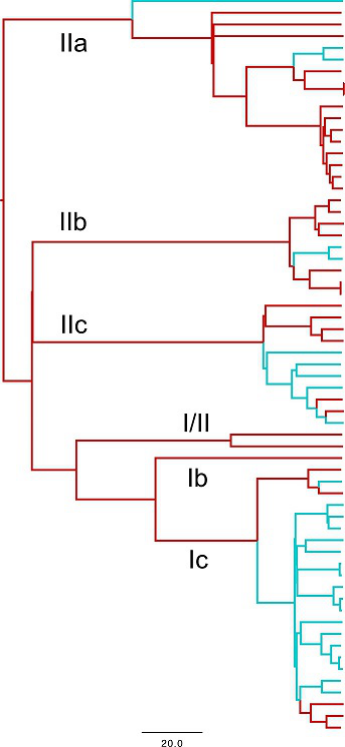
Maximum clade credibility tree. Predicted location of the animal isolates (*n*=63) as inferred by ancestral state reconstruction coloured red for England and Wales and blue for Scotland. Scale bar is in years.

All three major STEC O157 lineages and 5/6 sub-lineages were represented (Fig. 3). For all sub-lineages there was limited evidence of strain migration between nations with a high degree of strain segregation. Ancestral state reconstruction predicted only 6.7 [95 % highest posterior density (HPD): 4–9] migrations of STEC O157:H7 diversity between England and Scotland and 3.9 (95 % HPD: 1–8) migrations in the other direction (Fig. 3). From the maximum clade credibility tree, 67 % (*n*=6/9) of these transitions occurred at least 10 years previously and therefore direct inference of strain migration is uncertain. In contrast, there was evidence of recent within-nation transmission of strains between farms with two Scottish sub-lineage Ic strains three SNPs apart, two sub-lineage IIb strains on the English/Welsh borders three SNPs apart and two identical English sub-lineage IIa strains in the North West of England.

### Diversity of strain types in humans

Within the clinical cases in GB all major lineages and sub-lineages were represented (Table 1). Based on analysis of the WGS data from 684 isolates, clinical cases from Scotland were significantly more likely to be from sub-lineage Ic [50.9 vs 31.4 %, *P*<0.001, OR 2.2 (1.5–3.2)] whereas clinical cases from England and Wales were significantly more likely to be from sub-lineage IIb [14.9 vs 0.6 %, *P*<0.001, OR 29 (4.9–1161)] (Table 1).

Sub-lineage Ic clinical cases from Scotland were predominately PT21/28 [41.9 vs 24.9 %, *P*<0.001, OR 2.18 (1.47–3.21)] and PT32 [10.0 vs 4.5 %, *P*=0.012, OR 2.36 (1.13–4.80)]. Sub-lineage IIb clinical cases from England and Wales were predominantly PT8 [43.1 vs 33.8, *P*=0.042, OR 1.48 (1.01–2.20)] and PT1 [5.1 vs 0.63 %, *P*=0.010, OR 8.52 (1.38–352)] (Table 1).

A similar proportion of clinical infections were caused by an Stx2a+ strain in Scotland compared to England and Wales (59.6 vs 52.4 %, *P*=0.1247) for the time frame of this study (Fig. 3c). In Scotland, 85.4 % (*n*=82) of Stx2a+ strains were sub-lineage Ic, primarily PT21/28. In England and Wales, Stx2a+strains were distributed across several lineages and sub-lineages [Ic (58.8 %), IIb (20.4 %), I/II (9.1 %), Ia (4.4 %), IIa (5.5 %) and IIc (1.5 %)] (Table 1). Based on PT, the total number of different clinical strains in England and Wales was higher than in Scotland (Fig. 3a, b), but the diversity was similar with respect to the remaining diversity measures (Fig. 3a).

### Analysis of human lineages

Five SNP single linkage clusters (SLCs) have been used operationally in the UK to define isolates that are likely to be epidemiologically linked [6, 11, 35]. From the 684 clinical cases from England, Wales and Scotland, there were 82 clusters (two or more isolates), and 70 (85 %) of these contained isolates from only one of the two regions (Scotland or England and Wales), indicating that exposures were generally restricted at a national level. However, if larger clusters or outbreaks are examined, 57 % (4/7) have cases in both England and Wales and Scotland. Seven clusters (Table S3.1) contained more than five cases and were investigated epidemiologically as potential outbreaks during the time frame of this study. Three of these clusters were associated with the consumption of bagged salad of domestic origin, two in sub-lineage IIc and one in sub-lineage IIb. Four outbreaks occurred in England and Wales involving sub-lineage Ic strains, the exposure of which could be linked to food, animal or environmental exposure (Table S3.1).

**Fig. 4.**
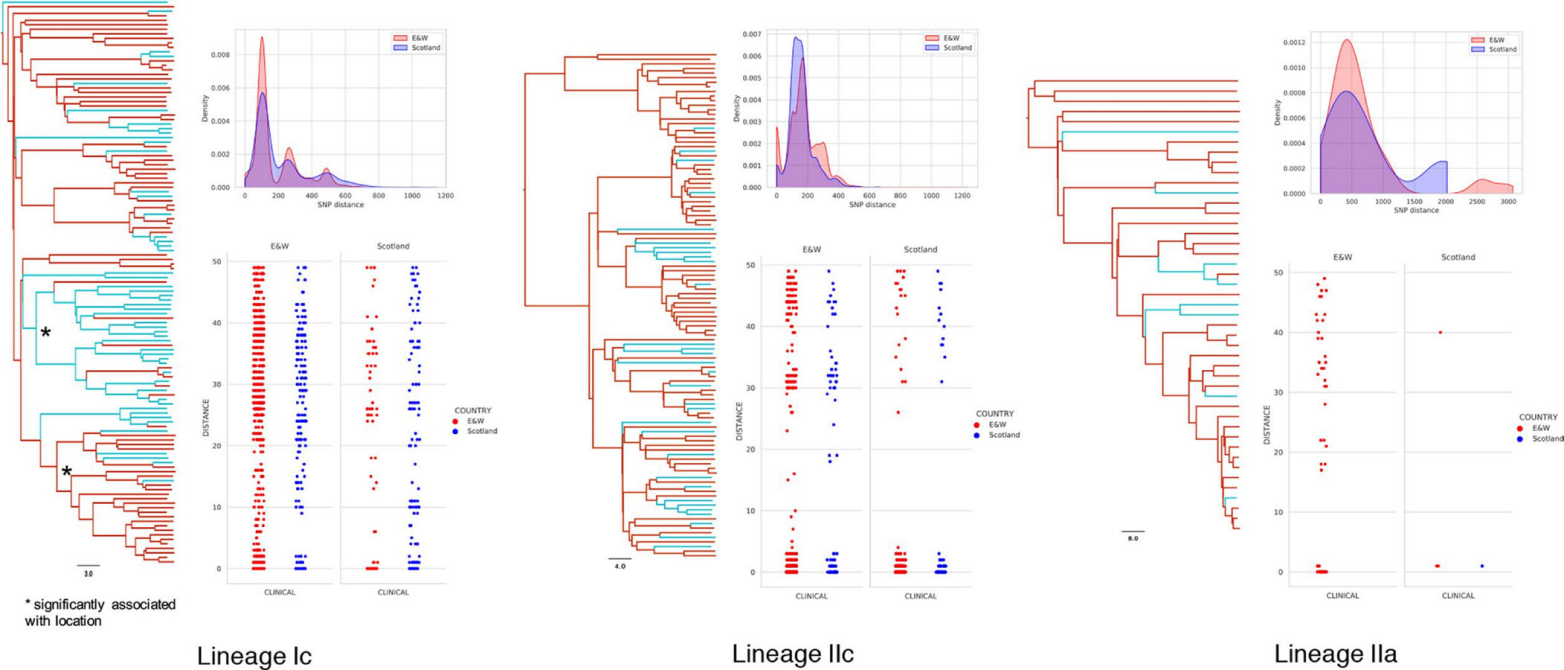
Maximum clade credibility tree. For the three major lineages in the study population (Ic, IIc, IIa), maximum clade credibility trees with predicted locations as inferred by ancestral state reconstruction coloured red for England and Wales and blue for Scotland (left). Clades significantly associated (*P*<0.05) with locations are highlighted with an asterisk. Probability density functions of the pairwise SNP distances between clinical isolates of the same location (top right). Scatterplot showing the pairwise SNP distances between each clinical isolate’s top 10 % closest matches delineated by location (bottom right). Scale bar is in years.

In contrast to the observed segregation in diversity of cattle isolates between Scotland and England and Wales, the human clinical isolates showed a greater degree of mixing. Examining location as a discrete trait on the phylogeny identified multiple migration events between locations for all main sub-lineages (Ic, IIc and IIa) (Fig. 4). For sub-lineage Ic the segregation between cattle isolates in England and Wales and Scotland can be explained by a single migration event, but for the clinical isolates approximately 14 % (30/216) of the bifurcations represent a transition in source location. For sub-lineage IIc, 13 % (25/194) of bifurcations represent a transition in source location whereas the cattle segregation between Scotland and England and Wales can be explained by three migration events. For sub-lineage IIa, 10 % (9/85) of bifurcations represent a transition in source location whereas the cattle segregation between Scotland and England and Wales can be explained by two migration events. Despite the high degree of mixing, within the Ic phylogeny two clades were identified, one associated with England and Wales and one with Scotland. For IIc and IIa, there were no significant associations between clade and location.

The genetic distances across the main sub-lineages (Ic, IIc, IIa) were calculated for the clinical isolates within and between Scotland and England and Wales. (Table 2, Fig. S3.1. S3.2, S3.3). For sub-lineages Ic and IIc the SNP distance distributions between clinical isolates as described by probability density functions were significantly different between England and Wales clinical isolates and Scottish clinical isolates, but this was not the case for sub-lineage IIa (although there were relatively low numbers for this analysis). Across the whole O157 population, the proportion of England and Wales isolates clustering with other England and Wales isolates (closest 10 % by SNP distance) was significantly enriched compared to the overall proportion in the study population (OR 5.23, *P*<0.05) but not significantly different for Scottish clinical isolates. Stratified by major sub-lineages, Ic isolates from both England and Wales and Scotland were more likely to be closer to an isolate from the same location than from a different location (England and Wales OR 2.31, *P*<0.05; Scotland OR 3.87, *P*<0.05) and this was also the case for sub-lineage IIc from England and Wales (OR 3.36, *P*<0.05) but not from Scotland (OR 1.14, *P*>0.05). In summary, sub-lineage Ic clinical isolates were more likely to be clustered by location while for for sub-lineage IIc strains this was only the case for England and Wales.

**Table 2.** The proportion of clinical isolates from England and Wales and Scotland delineated per lineage and overall compared to the cumulative proportion of isolates from the closest 10 % from each clinical (A) and cattle (B) isolate from each location

		England and Wales		Scotland	
Sub-lineage	Total	Closest 10%	OR	Closest 10%	OR
**(A) Human**					
IC	164 : 82	789 : 171	2.31*	61 : 118	3.87*
IIC	167 : 54	945 : 91	3.36*	275 : 79	1.14
IIA	52 : 10	50 : 0	0.0	3 : 1	1.73
All	508 : 160	4158 : 246	5.23*	696 : 204	1.14
**(B) Cattle**					
IC	164 : 82	25 : 2	6.25*	56 : 47	1.68*
IIC	167 : 54	10 : 1	3.23	1 : 8	24.7*
IIA	52 : 10	21 : 2	2.02	–	–
All	508 : 160	120 : 16	2.36	59 : 55	2.98

*Significant odds ratio (OR) calculated from Fisher’s exact test.

### Distribution of clinical cases with respect to cattle

Across all sub-lineages there was no significant separation between cattle and clinical isolates phylogenetically, suggesting the diversity captured in the GB cattle isolates can account for the diversity observed in domestically acquired O157 human infection in the same time period.

Comparing the cattle isolates to the closest 10 % of clinical isolates revealed that clinical isolates from Scotland were significantly (OR 2.98, *P*<0.05) more likely to cluster with Scottish cattle isolates while clinical isolates from England and Wales were more likely to cluster with cattle isolates from England and Wales (OR 2.36, *P*<0.05). This observation was consistent when stratifying by sub-lineage (Table 2).

## Discussion

Over the last two decades, the rate (per 100 000 population) of reported clinical cases of O157 in Scotland has been 2.5-fold higher than in England and Wales. This study has shown that for the period 2014–2015 Scottish clinical cases consisted of significantly more PT21/28 strains (sub-lineage Ic, Stx2a+) than England and Wales cases. While there were similar proportions of O157 strains with Stx2a+ associated with human infections in England and Wales compared with Scotland, in Scotland >80 % of the Stx2a+ strains were associated with sub-lineage Ic (PT21/28) as opposed to England and Wales where Stx2a+ strains were associated with various sub-lineages and alternative PTs (e.g. PT8, PT54) [5]. Strains producing Stx2a+, particularly those belonging to lineage Ic (PT21/28), are known to be associated with severe clinical outcomes, such as HUS [5] and sub-lineage IIb PT8 strains cause more severe disease when *stx2a* replaces *stx2c* [9]. Individuals infected with strains that cause more severe symptoms are more likely to present to primary healthcare settings and hence there was a higher number of reported clinical cases. Historical distributions of PTs for human infections in Scotland and England and Wales (Fig. S1.1B) show that while PT21/28 was the predominant strain in both countries for many years, the proportion of cases was always higher in Scotland.

The significantly higher representation of PT21/28 (sub-lineage Ic, Stx2a+) in Scottish clinical cases is mirrored in Scottish cattle. Despite similar overall prevalence of O157 in cattle between Scotland and England and Wales [18], Scottish cattle had a significantly higher proportion of strains encoding Stx2a, predominately made up of sub-lineage Ic PT21/28 (Stx2a+) strains which are associated with super-shedding and higher rates of cattle-to-cattle transmission [36–38]. PT21/28 was first detected in Scotland in 1993 and appears to have found a niche in the animal reservoir, especially in association with Scottish beef cattle in Northern Scotland. The relatively indigenous relationship of PT21/28 with Northern Scotland is not understood. While we can speculate that local maintenance of specific strains could be explained by local breeding practices combined with intermittent exchange with endemic wildlife or other farmed species, the original ‘settlement’ of strains is not clear and such distributions could be relatively random due to original stocking decisions. Geographical variation in O157 sub-types has been shown in other countries, such as Sweden where the rate of human O157 infection is low but serious pathology is associated with lineage I/II clade 8 Stx1a/2a strains which are predominately found in cattle only in the South East part of the country, and in this case these strains are associated with infections nationally [39]. It is possible that drivers such as cattle breed, husbandry system, climate, local flora and fauna could all be important for localization. However, research over the last two decades on O157 in Scottish cattle has not revealed a consistent association other than housing, farm size and recent movements onto the farm [21, 40–42]. This observation was confirmed by a 2010 modelling study [42] of Scottish farms that revealed a flat risk profile for Scottish farms suggesting that there were no strong associations despite many potential risk factors examined. This may be the result of the overall transient nature of O157 on farms [40, 43]. However, one study looking at farms that had been re-sampled after 2 years found similar strains in the new cattle suggesting a possible environmental reservoir [44]. Although only 20 % of Scottish farms harbour O157 at any time, 80 % will be positive over the course of the year [40].

It is interesting to note that the main sub-lineages in the UK have opposing phage resistance profiles; in other words, sub-lineage Ic is primarily resistant to T4 phage whereas IIc/b are resistant to T7 phage (and visa versa), at least in relation to sensitivities established within the typing phage panel [45]. It has been proposed that this may indicate alternative niches/lifestyles for the two sub-types of *E. coli* O157 and may also relate to localization of predating phage populations [46], even though both O157 subtypes are found in cattle. Alternatively, as discussed by the authors, negative frequency-dependent selection (NFDS) would be at play where population complexity is maintained as larger populations end up being predated upon. As more information comes from metagenomic studies, it will be interesting to see how localized and persistent specific phage populations can be and their relationship with susceptible hosts. Our study focused only on *E. coli* O157, and the wider diversity of bacteria susceptible to T4 and T7 phage needs to be taken into account.

Our study has focused on cattle as the primary source of O157 infection in the UK, either directly or indirectly. It should be noted that analysis of the WGS data indicates the majority of *E. coli* O157 diversity in human cases is captured by isolates we have sampled from cattle, indicating that there does not need to be another reservoir maintaining a different set of strain sub-types of threat to human health. However, we appreciate that other ruminants, especially sheep, are also a reservoir for human infection [15]. A recent study of the Scottish deer population has shown that although the prevalence of O157 was very low (<1 %), PT21/28 Stx2a+ strains were found including one with a direct link to a human outbreak [47]. Sheep and cattle are often co-grazed and deer are usually free to roam across farms in GB. As such there are ample opportunities for exchange of strains amongst all ruminants, either directly or from the shared environment. The subtypes isolated from human cases linked to exposure to sheep, lambs and deer, are also common in cattle [12, 47]. As such, the relative contribution of different ruminant species to the overall burden of infection is therefore difficult to define especially given the fact that sheep and cattle are farmed in the same geographical areas in the UK [48].

Ancestral state reconstruction from WGS data for cattle isolates indicated there was little mixing between Scotland and England and Wales supporting the concept that the subtypes were relatively localized. This localization results in different national distributions of strains in cattle with the potential to cause serious disease and, as proposed, could contribute to the higher frequencies of human O157 infection in Scotland compared to England and Wales. Our WGS analysis also found clear evidence of human cases being more locally derived, and clinical isolates from one nation were more likely to cluster with cattle isolates from that nation. Sub-lineage Ic clinical isolates, which are primarily PT21/28, were the most likely to be restricted to Scotland but this association breaks down when larger outbreaks were analysed. This makes sense in light of the fact that some of the larger outbreaks involve products, such as bagged salads, that have a multi-national distribution.

The simplest proposal for the outbreaks being more likely to be nationally restricted is infection from the local environment or contaminated food that has a local source. The risk of environmental exposure will vary in importance depending on location (i.e. rural setting) and nature of the visit (business vs holiday) with visitors to rural areas at more risk of STEC infection [49]. Compared to England and Wales, Scotland has a higher level of rurality and a higher cattle-to-human ratio (Table S4.1; Fig. S4.1). In addition, the laws of access in Scotland mean that there are potentially more opportunities for interaction between humans and cattle and/or their environment. Cattle density, sheep density and private water supply were associated with sporadic (non-outbreak-related) PT21/28 but not PT8 in England [48]. Other outbreaks of PT21/28 caused by exposure to a contaminated environment have been described [15]. Carriage levels (super-shedding) and transmission rates have been shown to be higher in cattle carrying PT21/28 [30–32], resulting in higher levels in the environment. Halliday *et al*. [50] found an association between cattle carriage of PT21/28, farms in northern Scotland and private water supply indicating that PT21/28 can exist in the environment and pose a risk to public health. On average, the risk of acquiring O157 was estimated to be 100 times greater when visiting a pasture compared with eating a burger [51]. Taken together, the intersection of higher shedding of some strain types, especially Stx2a+, and an increased chance of local exposure due to Scotland’s rurality probably acts in combination to increase the incidence of human disease. To our knowledge husbandry and abattoir practices are equivalent across the UK, with no obvious differences in processing methods that could explain the difference. Therefore, from a public health perspective, environmental and animal contact remain important risk factors for O157 infection [52].

We propose that the higher rate of O157 clinincal cases in Scotland is a result of the nationally high level of Stx2a+ *E. coli* O157 strains in the Scottish cattle population combined with more opportunities for local exposure through the environment and local food consumption. Local transmission will vary depending on exposure routes, while people and goods move freely within and between Scotland, England and Wales. Through both travel and food networks, people become exposed to strains that are not endemic to where they usually live which dampens the signal from local infections for human cases.

For public health, the safest approach may be to consider all farms a source of O157. It is important to point out that the presence of O157 on a farm is not an indication of poor animal husbandry, as this organism is a ubiquitous commensal in the gastrointestinal tract of cattle. As such, food safety must be controlled by ensuring appropriate transport to the abattoir, good practice at the abattoir, pre- and post-slaughter, and within the subsequent supply chain up to and including the point of food preparation. Although outbreaks associated with consumption of contaminated beef still occur, the frequency has decreased in GB over the last three decades [53], reflecting concerted efforts by the farming and meat manufacturing industry to improve practices and maintain high standards. It remains a fact that we have life-threatening *E. coli* O157 strains indigenous in UK cattle. While we continue to develop interventions such as vaccines, phage and probiotics and feed approaches that can be used on farm to reduce the threat to human health from all forms of transmission, education and awareness remain the main tools to reduce the risk of human infection.

## Supplementary Data

Supplementary material 1

Supplementary material 2
