# Supplementary material for: Analysis of Escherichia coli O157 strains in cattle and humans between Scotland and England & Wales: implications for human health
Source: Microb Genom. 2023 Sep 6;9(9):001090. doi: 10.1099/mgen.0.001090 (PMC10569735; doi:10.1099/mgen.0.001090)
Supplement: Supplementary material 1 [file mgen-9-1090-s001.pdf]

Supplementary material 1. Background

1.0 Temporal trends in Scotland and England & Wales

1.1 Rate (per 100,000 population)

Historical data was obtained from annual reports from Public Health England (PHE) [1], The Scottish *E. coli* / STEC Reference Laboratory (SERL) [2] and Public Health Scotland (PHS, formerly Health Protection Scotland (HPS)) [3] for the total number of reported human clinical cases (Fig. S1.1A) and the distribution of phage types (PT) from 1998 to 2019 (Fig. S1.1B). We chose to start with the year 1998 as there was a large outbreak in Central Scotland in 1996/1997 which inflated the case numbers.

The yearly count data includes outbreak and travel-related cases. The case data was analyzed using Poisson regression analysis of the number of human reported clinical cases in Scotland and England & Wales 1998-2019 adjusted for population size (mid-year population size [4]). The model was fit using a generalised linear model with a Poisson distribution fitted to count data, offset by the logarithm of population size, to identify differences in the rates of O157 infection across time (year: 1998-2019; p=0.001) and location (Scotland vs England & Wales; p<0.001) (Table S1.1). The interaction was not significant so it was removed from the model (p=0.9626). The overall rate of O157 is declining in the UK (Fig. S1.1A). There was a significant difference between the rate of O157 in Scotland (4.0 (3.6-4.3)) and England and Wales (1.6 (1.5-1.7)). Analyses were carried out using Proc Glimmix in SAS version 9.4 (SAS Institute Inc., Cary, NC)

Table S1.1 Results from the Poisson regression analysis

Variable	Estimate	se	p
intercept	-10.86	0.067	<0.001
Location			
Scotland	0.910	0.062	<0.001
E&W	-	-	
Year	-0.017	0.0049	0.001

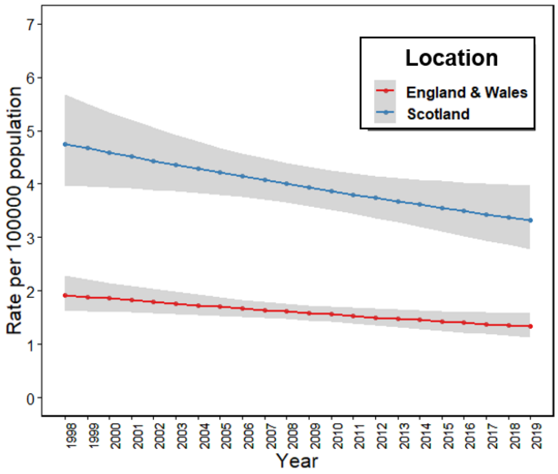

Fig. S1.1A Rate per 100,000 population of reported clinical *E. coli* O157 cases in Scotland and England & Wales between 1998 and 2019.

1.2 Proportion of cases for different Phage Types

Fig. S1.1B shows the proportion of cases of the predominant phage types (PT) In England & Wales and Scotland (1994-2019). This includes all samples submitted to SERL, PHE and PHS over a given calendar year (1994-2019). Travel-related cases and outbreak cases are included. The graph demonstrates

- the high proportion of cases of PT21/28 in Scotland, especially between 1997 (when it replaced PT2) to 2011 (when the proportion of PT8 cases increased to the same level of PT21/28).
- Strain replacement of PT2 by PT21/28 and the decline of PT21/28 and increase of PT8 in both Scotland and England & Wales

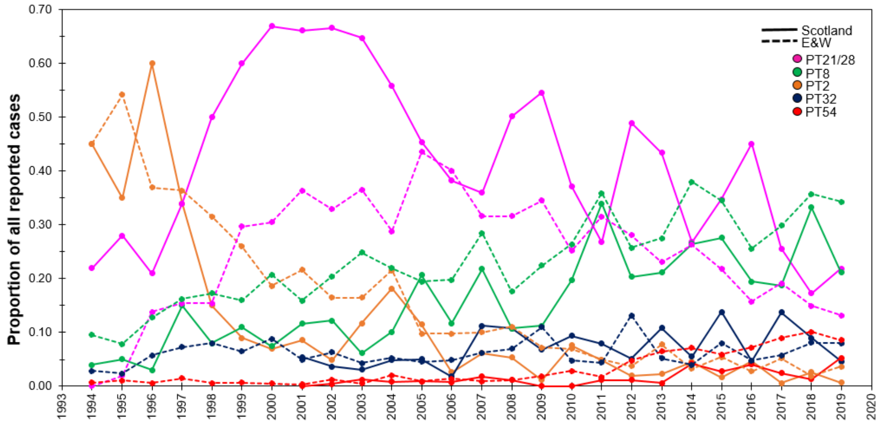

Figure S1.1B Proportion of cases of the predominant phage types (PT) in England & Wales (dashed lines) and Scotland (solid lines).

2.0 Spatial Regions of Scotland, England & Wales

In Scotland, in previous publications we have defined 6 Animal health Districts (AHDs) (Fig. S1.2A) (Pearce et al., 2009; Henry et al., 2017) including the following: Highland, Islands, North East, Central, South East, South West. In England & Wales spatial regions were defined using the Nomenclature of Units for Territorial Statistics (NUTS) (Fig. S1.2B). NUTS 1 regions for England & Wales include: North east, North West, Yorkshire, East Midlands, West Midlands, East of England, London (no data), South east, South West, West. Spatial regions were defined to ensure at least 5 farms in each region to preserve confidentiality. European Parliament, Council of the European Union. Regulation (EC) No 1059/2003 of the European Parliament and of the Council of 26 May 2003 on the establishment of a common classification of territorial units for statistics (NUTS). Official Journal of the European Union L154 2003; 46:1

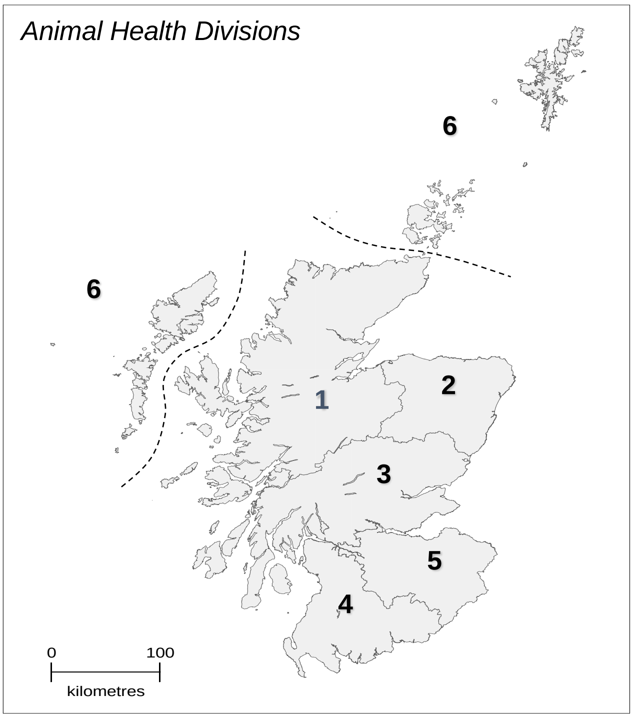

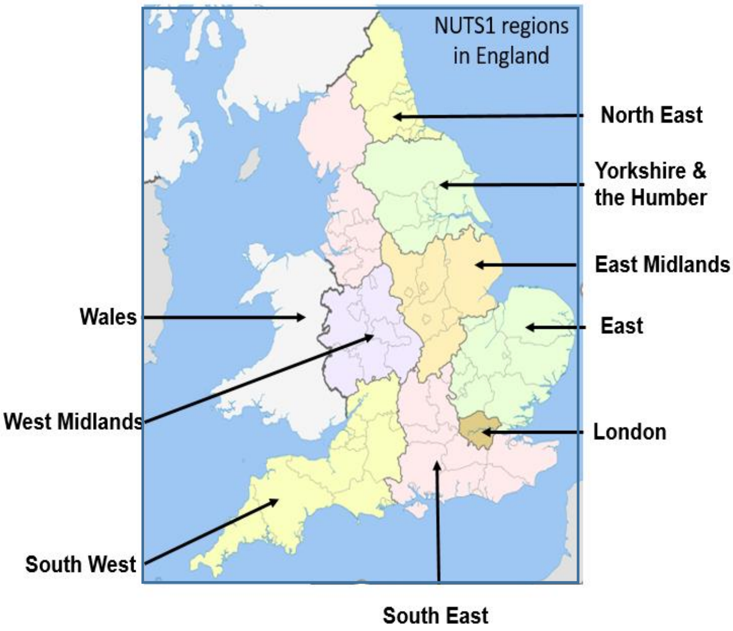

Figure S1.2 A. In Scotland spatial regions were defined using the 6 Animal health Districts (AHDs) in Scotland (from Pearce et al., 2009) including the following: Highland (1), Islands (6), North East (2), Central (3), South East (5), South West (4). **B.** In England & Wales spatial regions were defined using the Nomenclature of Units for Territorial Statistics (NUTS) (from File: English regions 2009.svg - Wikipedia). NUTS 1 regions for England & Wales include: North east, North West, Yorkshire, East Midlands, West Midlands, East of England, London, South east, South West, West.

References

[1] Public Health England. https://www.gov.uk/government/organisations/public-health-england)
[2] The Scottish *E. coli*/STEC Reference Laboratory. https://www.edinburghlabmed.co.uk/Specialities/reflab/ecoli/Pages/default.aspx
[3] Health Protection Scotland. https://www.hps.scot.nhs.uk
[4] National records of Scotland, mid-year population estimates. https://www.nrscotland.gov.uk/statistics-and-data/statistics/statistics-by-theme/population/population-estimates/mid-year-population-estimates

Appendix 2: Associations among typing methods PT, stx subtype, lineage (WGS)

Fig. S2.1 shows how Lineage, PT, and stx subtype are associated within this study. Diagram was constructed using Interactive Tree Of Life (iTOL) program [1] and included all clinical (n=684) and cattle (n=113) isolates sequenced within this study.

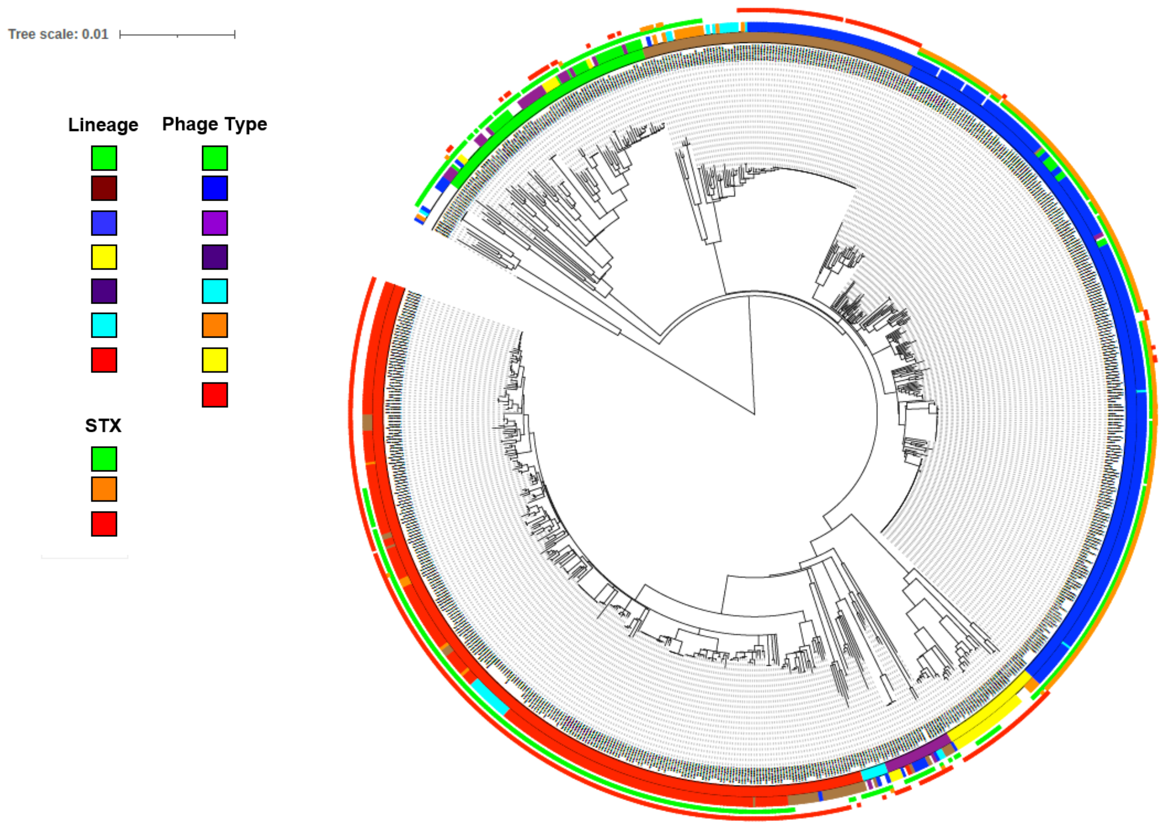

Figure S2.1 Association among Lineage (ring 1), PT (ring 2), and stx subtype (ring 3-5) for the isolates in this study. Rings are defined from the inner circle to out.

References

[1] Letunic I, Bork P. Interactive Tree Of Life (iTOL): an online tool for phylogenetic tree display and annotation. *Bioinformatics* 2007; 23(1): 127–8. Available from: http://www.ncbi.nlm.nih.gov/pubmed/17050570

Appendix 3: Clustering (WGS)

3.1 Outbreaks

Five SNP single linkage clusters (SLC) have been used operationally in the UK to define isolates that are likely to be epidemiologically linked [1]. From the 684 clinical cases from England, Wales and Scotland, there were 82 clusters (2 or more isolates) and 70 (85%) of these contained isolates from only one of the two regions (Scotland or England & Wales), indicating that exposures were generally restricted at a national level.
The picture is different if larger clusters or outbreaks are examined with 57% (4/7) of having cases in both England & Wales and Scotland. The seven clusters (Table S3.1) contained greater than 5 cases and were investigated epidemiologically as potential outbreaks over the time frame of this study. Three of these clusters were associated with the consumption of bagged salad of domestic origin, two in lineage IIc (Fig. S3.1) and one in lineage IIb (Fig. S3.2). Four outbreaks occurred in E&W involving lineage Ic strains in the study period (Fig. S3.3). This included OB4 associated with a butcher's shop and linked back to a cattle farm in the North East [2], OB5 associated with slaw consumption [3], OB6 associated with a private water supply in the North West [4], and OB7 associated with the consumption of raw drinking milk [5] sourced from the South West of England. In all four outbreaks the exposure could be linked to food, animal, or environmental exposure.

Table S3.1 Outbreak during time frame of study. E&W, England & Wales.

Outbreak	Sublineage	Number of Cases		Source
		E&W	Scotland	
OB1	IIc	51	10	Domestic Bagged Salad[6]
OB2	IIc	18	6	Domestic Bagged Salad[6]
OB3	IIb	46	0	Domestic Bagged Salad[6]
OB4	Ic	13	1	Butchers[2]
OB5	Ic	14	0	Slaw[3]
OB6	Ic	5	3	Private Water Supply[4]
OB7	Ic	9	0	Raw drinking milk[5]

References

[1] Dallman T, Ashton P, Schafer U, Jironkin A, Painset A, Shaaban S, Hartman H, Myers R, Underwood A, Jenkins C, Grant K. SnapperDB: a database solution for routine sequencing analysis of bacterial isolates. *Bioinformatics* 2018; 34(17): 3028-9. DOI: 10.1093/bioinformatics/ bty212
[2] Wilson D, Dolan G, Aird H, Sorrell S, Dallman TJ, Jenkins C, Robertson L, Gorton R. Farm-to-fork investigation of an outbreak of Shiga toxin-producing *Escherichia coli* O157. *Microb Genom* 2018; 4(3) DOI:10.1099/mgen.0.000160
[3] Byrne L, Adams N, Glen K, Dallman TJ, Kar-Purkayastha I, Beasley G, Willis C,

Padfield S, Adak G, Jenkins C. Epidemiological and Microbiological Investigation of an Outbreak of Severe Disease from Shiga Toxin-Producing *Escherichia coli* O157 Infection Associated with Consumption of a Slaw Garnish. *J Food Prot* 2016; 79(7): 1161-1168. DOI: 10.4315/0362-028X.JFP-15- 580 PMID: 27357035
[4] Rowell S, King C, Jenkins C, et al. An outbreak of Shiga toxin-producing *Escherichia coli* serogroup O157 linked to a lamb-feeding event. *Epidemiol Infect* 2016; 144(12): 2494-2500. DOI:10.1017/S0950268816001229.
[5] Butcher H, Elson R, Chattaway MA, Featherstone CA, Willis C, Jorgensen F, Dallman TJ, Jenkins C, McLauchlin J, Beck CR, Harrison S. Whole genome sequencing improved case ascertainment in an outbreak of Shiga toxin-producing *Escherichia coli* O157 associated with raw drinking milk. *Epidemiol Infect*. 2016; 144(13): 2812-2823. DOI:10.1017/S0950268816000509.
[6] Sinclair C, Jenkins C, Warburton F, Adak GK, Harris JP. Investigation of a national outbreak of STEC *Escherichia coli* O157 using online consumer panel control methods: Great Britain, October 2014. *Epidemiol Infect* 2017; 145(5): 864-871. DOI:10.1017/S0950268816003009. 66.

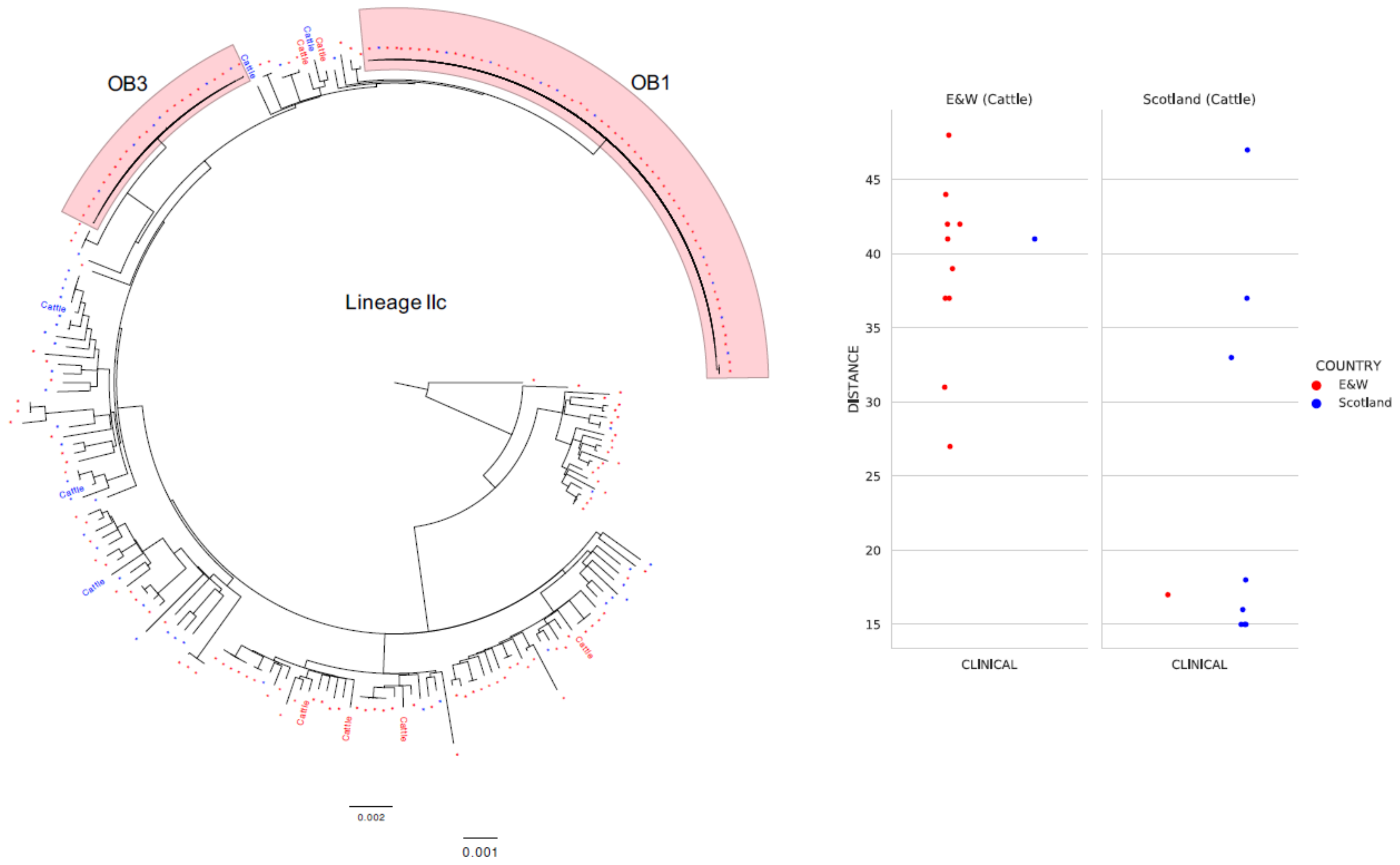

Fig. S3.1 Maximum likelihood phylogenies of clinical and cattle isolates from lineage IIC. Taxa are labelled 'cattle' or '*' for clinical isolates and coloured red for England & Wales and blue for Scotland (right). Scatterplot showing the pairwise SNP distances between each cattle isolate and the closest clinical matches delineated by country (left). OB1: Outbreak 1; OB3: outbreak 3.

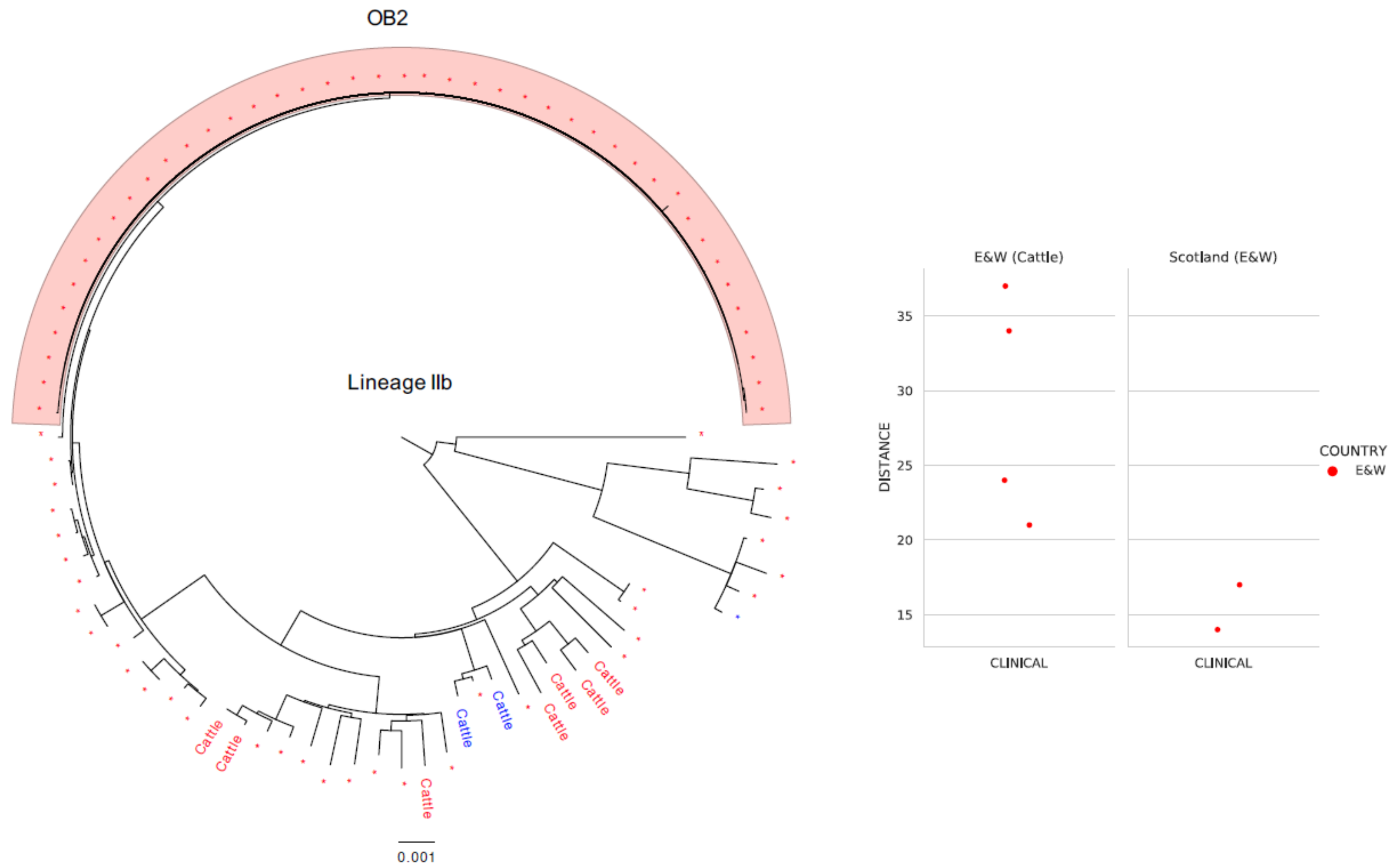

Fig. S3.2 Maximum likelihood phylogenies of clinical and cattle isolates from lineage IIB. Taxa are labelled ‘cattle’ or ‘*’ for clinical isolates and coloured red for England & Wales and blue for Scotland (right). Scatterplot showing the pairwise SNP distances between each cattle isolate and the closest clinical matches delineated by country (left). OB2: Outbreak 2.

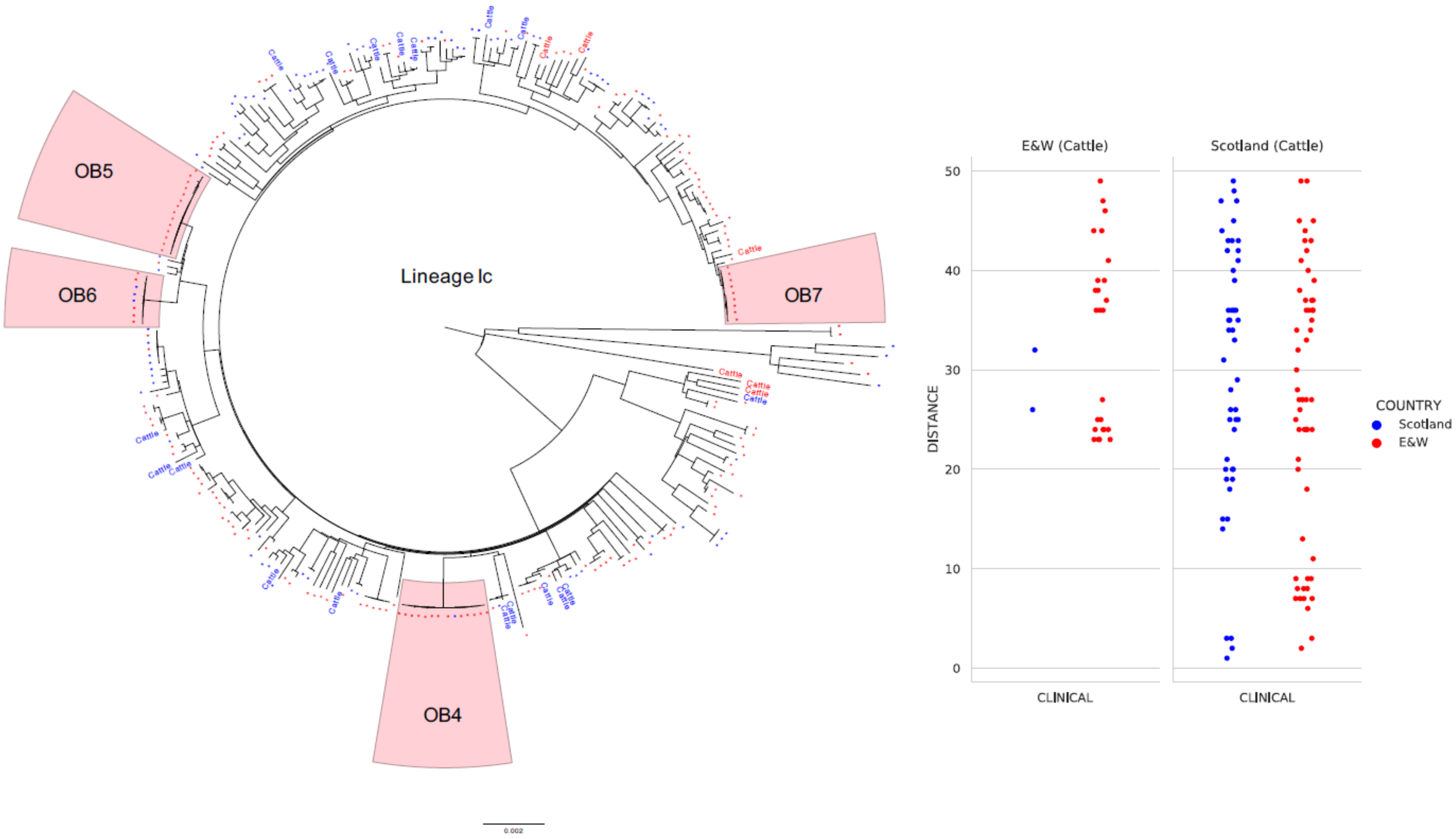

Fig. S3.3 Maximum likelihood phylogenies of clinical and cattle isolates from lineage IC. Taxa are labelled 'cattle' or '*' for clinical isolates and coloured red for England & Wales and blue for Scotland (right). Scatterplot showing the pairwise SNP distances between each cattle isolate and the closest clinical matches delineated by country (left). OB4: Outbreak 4; OB5: Outbreak 5; OB6: Outbreak 6; OB7: Outbreak 7.

Supplementary material 4: Data Tables for Figure 1 in main manuscript

Table S4.1 A&B contain the data associated with Fig. 1 in the main manuscript. In Scotland, counts of Phage types and presence/absence of *E. coli* O157 is expressed for the 6 Animal health Districts (AHDs) (Fig. S1.2A) as done historically (Pearce et al., 2009; Henry et al., 2017) including the following: Highland, Islands, North East, Central, South East, South West. In England & Wales spatial regions were defined using the Nomenclature of Units for Territorial Statistics (NUTS) (Figure S4.1B). NUTS 1 regions for England & Wales include: North east, North West, Yorkshire, East Midlands, West Midlands, East of England, London (no data), South east, South West, West. Phage types (PT) were grouped as follows: PT1, PT32, PT4, PT2, PT54, PT8, PT21/28 and PT Other (other includes the following PTs: 14, 31, 34, RDNC). Spatial regions were defined to ensure at least 5 farms in each region to preserve confidentiality. European Parliament, Council of the European Union. Regulation (EC) No 1059/2003 of the European Parliament and of the Council of 26 May 2003 on the establishment of a common classification of territorial units for statistics (NUTS). Official Journal of the European Union L154 2003; 46:1

Table S4.1. Data used in Figure 1 of the main manuscript for (A) Scotland and (B) England & Wales.

A. Scotland

Region	Region	PT21/28	PT8	PT54	PT2	PT4	PT32	PT1	PTOther	pos	neg	total
1	South West	2	1	0	0	0	1	0	0	4	19	23
2	South East	1	1	0	0	2	0	0	1	4	13	17
3	Highland	5	0	0	0	0	0	0	0	5	14	19
4	North East	4	1	0	0	0	0	0	0	5	14	19
5	Central	0	1	0	0	0	0	0	2	3	12	15
6	Islands	4	0	0	0	0	0	0	0	4	12	16

B. England & Wales

Region	NUTS1	location	PT21/28	PT8	PT54	PT2	PT4	PT32	PT1	PTOther	pos	neg	total
1	UKC	North east	1	0	0	0	1	0	0	0	2	4	6
2	UKD	North West	0	0	0	0	0	1	0	2	3	9	12
3	UKE	Yorkshire	0	0	0	1	0	0	0	0	1	11	12
4	UKF	East Midlands	0	0	0	0	0	0	0	1	1	9	10
5	UKG	West Midlands	1	3	2	0	2	0	2	1	9	16	25
6	UKH	East of England	0	0	1	0	0	0	0	0	1	4	5
7	UKI	London	0	0	0	0	0	0	0	0	0	0	0
8	UKJ	South East	0	0	0	1	0	1	0	1	3	13	16
9	UKK	South West	0	1	5	0	0	1	0	1	7	23	30
10	UKL	Wales	1	2	0	0	2	0	2	1	7	36	43

References

[1] Pearce MC, Chase-Topping ME, McKendrick IJ, Mellor DJ, Locking ME, *et al.* Temporal and spatial patterns of bovine *Escherichia coli* O157 prevalence and comparison of temporal changes in the patterns of phage types associated with bovine shedding and human *E. coli* O157 cases in Scotland between 1998–2000 and 2002–2004. *BMC Microbiol* 2009;9:276–289. doi: 10.1186/1471-2180-9-276

[2] Henry MK, Tongue SC, Evans J, Webster C, McKendrick IJ, *et al.* British *Escherichia coli* O157 in Cattle Study (BECS): to determine the prevalence of *E. coli* O157 in herds with cattle destined for the food chain. *Epidemiol Infect* 2017;145(15):3168–3179. doi:10.1017/S095026881700215i

Supplementary material 5: Data Tables: Strain composition

Table S5.1 Complete list of all possible stx subtypes for each phage type. There is a tick (✓) in the cell if the strain was found in cattle or human clinical cases from Scotland or England or Wales.

		Scotland		England & Wales	
**Phage Type**	**Stx subtype**	**Cattle**	**Human**	**Cattle**	**Human**
21/28	2a	✓	✓	✓	✓
	2a+2c	✓	✓		✓
	2a+2c+1a	✓			
	2a+1a				
	2c		✓		
	2c+1a				
	1a				
	negative				
8	2a				✓
	2a+2c		✓		✓
	2a+2c+1a		✓		✓
	2a+1a				✓
	2c		✓		✓
	2c+1a	✓	✓	✓	✓
	1a	✓	✓		✓
	negative				
54	2a				
	2a+2c		✓	✓	
	2a+2c+1a				
	2a+1a				
	2c		✓	✓	✓
	2c+1a		✓		✓
	1a				
	negative				
2	2a		✓		✓
	2a+2c		✓	✓	✓
	2a+2c+1a				
	2a+1a				
	2c			✓	✓
	2c+1a				
	1a				
	negative				
32	2a		✓		✓
	2a+2c		✓		✓
	2a+2c+1a				
	2a+1a				✓
	2c	✓	✓	✓	✓
	2c+1a			✓	✓
	1a				
	negative			✓	
4	2a		✓		✓
	2a+2c			✓	✓
	2a+2c+1a				✓
	2a+1a				
	2c	✓		✓	✓
	2c+1a		✓		✓
	1a				
	negative				

Table S5.1 continued. Complete list of all possible stx subtypes for each phage type. There is a tick (✓) in the cell if the strain was found in cattle or human clinical cases from Scotland or England or Wales.

		Scotland		England & Wales	
Phage Type	Stx subtype	Cattle	Human	Phage Type	Stx subtype
14	2a		✓		✓
	2a+2c			✓	✓
	2a+2c+1a				✓
	2a+1a				
	2c	✓		✓	✓
	2c+1a		✓		✓
	1a				
	negative				
34	2a				✓
	2a+2c			✓	✓
	2a+2c+1a				
	2a+1a				✓
	2c	✓	✓	✓	✓
	2c+1a				✓
	1a				
	negative	✓		✓	
31	2a				
	2a+2c				
	2a+2c+1a				
	2a+1a				
	2c			✓	
	2c+1a				
	1a				
	negative				
1	2a				✓
	2a+2c		✓		✓
	2a+2c+1a				
	2a+1a				✓
	2c				✓
	2c+1a				
	1a				
	negative			✓	
